# A Rare Case of Necrotizing Pneumonia and Pleural Empyema Secondary to Transdiaphragmatic Extension and Perforation of a Pyogenic Liver Abscess

**DOI:** 10.7759/cureus.70273

**Published:** 2024-09-26

**Authors:** Jorge Mendes, Miguel G Santos, Gonçalo Dias, Ricardo Marinho, Fernando Henriques

**Affiliations:** 1 Intensive Care Unit, Centro Hospitalar de Leiria, Leiria, PRT; 2 Department of Critical Care Medicine, Centro Hospitalar de Leiria, Leiria, PRT; 3 Department of General Surgery, Centro Hospitalar de Leiria, Leiria, PRT

**Keywords:** emergency medicine, klebsiella pneumoniae (kp), liver abscess, necrotizing pneumonia, pleural empyema, pyogenic abscess

## Abstract

This article presents a rare case of an elderly patient with diabetes and hypertension who developed a primary pyogenic liver abscess (PLA) that subsequently disseminated to the lungs by contiguity, resulting in diaphragmatic perforation complicated by necrotizing pneumonia, hepatobronchial fistula, and pleural empyema. In this case, percutaneous drainage of the PLA was unsuccessful, necessitating surgical intervention, which confirmed the diaphragmatic perforation. *Klebsiella pneumoniae* was isolated from the liver abscess samples sent for microbiological analysis, while blood cultures were negative. Despite extensive local infection and systemic dissemination consistent with invasive liver abscess syndrome, as well as progression to septic shock requiring intensive care unit admission, the patient achieved a gradual yet full recovery and ultimately returned to an active daily life. This was only possible due to the effective control of the infectious focus, combined with appropriate antibiotic therapy and supportive measures.

## Introduction

Pyogenic liver abscesses (PLAs) are collections of pus within the liver caused by infection of the hepatic parenchyma by bacteria, leading to a local inflammatory reaction and cellular destruction [1]. This is a rare condition, with an incidence of 1.1 to 2.3 per 100,000 inhabitants, higher in Asian countries, where it can reach 17.6 cases per 100,000 inhabitants [1,2]. Initially described as a disease of young people, there is now a growing incidence among the elderly [3]. The etiology of PLAs can vary, although in Europe, biliary pathology is one of the most common causes [1,4], followed by cryptogenic abscesses caused by Escherichia coli, Staphylococcus aureus, and Streptococcus. On the other hand, in Southeast Asian countries, the most common cause is cryptogenic abscess due to Klebsiella pneumoniae.

PLAs are a life-threatening condition, with mortality reaching 80% in the first half of the 20th century [1]. While there has been a significant reduction in mortality to the present day, it remains notably high, ranging from 8% to 31% [5]. Contributing to the significant reduction in morbidity and mortality are advances in imaging techniques, particularly CT and ultrasound, the development of antibiotics, and enhancements in surgical techniques, including minimally invasive procedures and percutaneous drainage [1,5]. The high mortality rate may be associated with the increasing prevalence of conditions such as diabetes mellitus, biliary tract diseases, including neoplasms, and delayed diagnosis [5]. Patient symptoms can be varied but are often nonspecific, which may contribute to diagnostic delays. Fever, abdominal pain, particularly in the right upper quadrant, asthenia, fatigue, nausea, and vomiting, are the most common symptoms, making early diagnosis of PLAs a challenge [5,6].

## Case presentation

A 76-year-old man with a history of overweight, hypertension, type 2 diabetes mellitus, controlled with oral medication, and a daily alcohol intake of approximately 8 grams presented to the Emergency Department with complaints of asthenia, anorexia, and right upper quadrant abdominal pain, persisting for more than a week. Three days prior to admission, he experienced chest pain, fever, and progressively worsening dyspnea.

**Figure 1:**
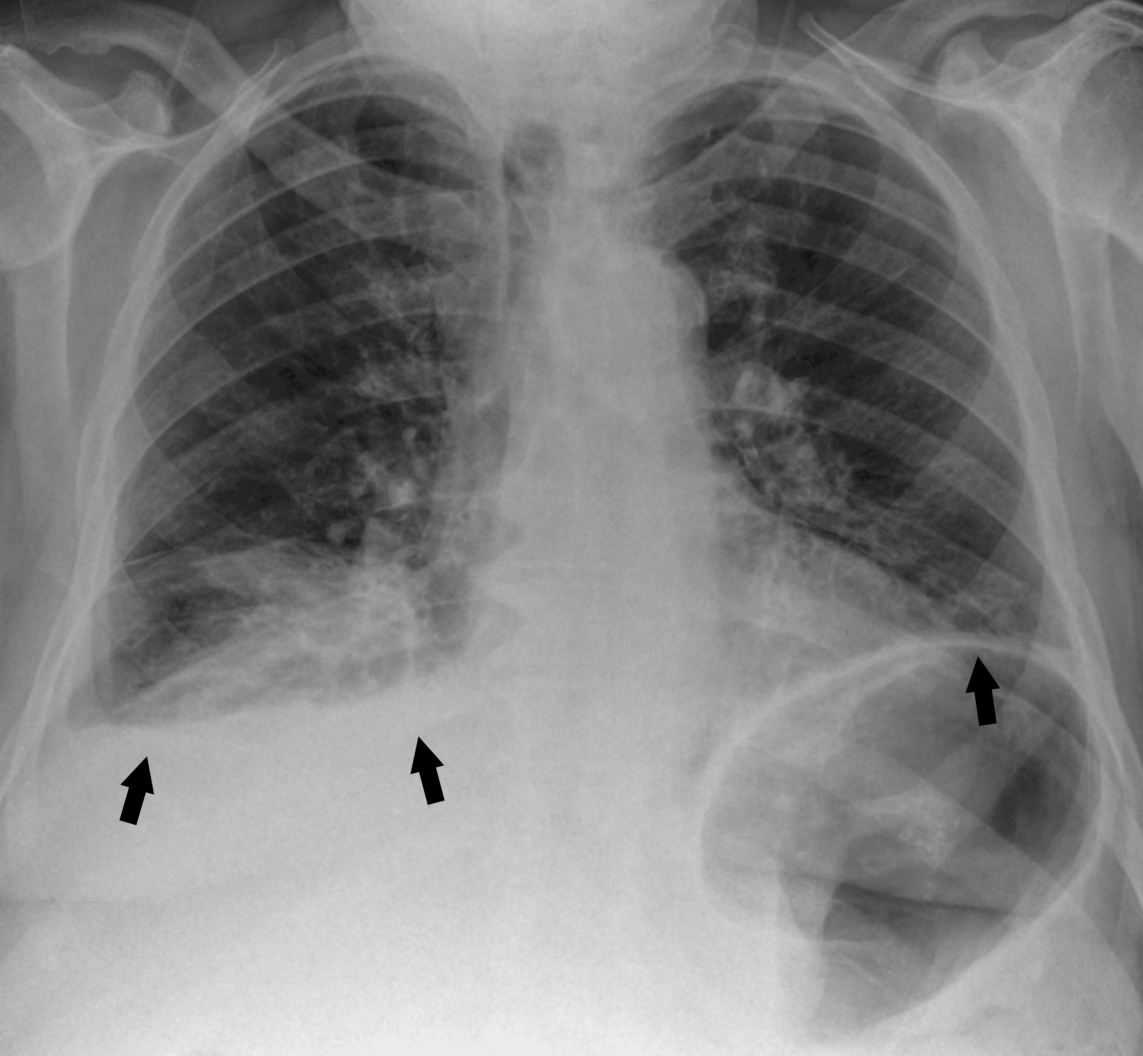
Patient's chest X-ray upon Emergency Department admission showing bilateral infiltrates (arrows), predominantly on the right side, consistent with pneumonia.

Upon admission to the Emergency Department, he had signs of respiratory distress, low oxygen saturation, and fever. Arterial blood gas analysis revealed a pH of 7.47, pO2 of 49 mmHg, and pCO2 of 28 mmHg, with normal lactate levels. Blood tests, including blood cultures, were performed. A nasal swab was collected to test for SARS-CoV-2, influenza A and B, respiratory syncytial virus, and urinary antigens for Streptococcus pneumoniae and Legionella, all of which were negative. The most significant blood test results are presented in Table 1. The chest X-ray showed bilateral infiltrates consistent with community-acquired pneumonia (Figure 1), so the patient was treated with amoxicillin/clavulanic acid and clarithromycin and admitted to the Internal Medicine ward. Two days later, the Intensive Care Unit (ICU) team was called due to significant clinical deterioration following an episode of frank hemoptysis. The patient exhibited clear signs of respiratory distress and severe cardiovascular dysfunction. Arterial blood gas analysis under a high-concentration oxygen mask showed a pH of 7.28, pO2 of 60 mmHg, pCO2 of 51 mmHg, bicarbonate of 19.1 mmol/L, and lactate of 3.5 mmol/L. During intubation, blood and thick purulent material were found in the airway. Blood tests, including microbiological studies, were repeated, with the most significant results presented in Table 1.

**Table 1:** Results of blood tests performed upon admission to the Emergency Department and upon admission to the ICU. AST: Aspartate transferase; ALT: alanine transaminase; ALP: alkaline phosphatase; GGT: gamma-glutamyl transferase; LDH: lactate dehydrogenase; CK: creatine kinase.

Blood analysis	Hospital admission	ICU admission	Normal range/unit
Leukocytes	15.7	22.1	4.0 – 10.0 /10^3/µL
Neutrophils	12.2	18.9	1.8 – 8.0 /10^3/µL
Hemoglobin	12.6	11.9	13.0 – 17.7 /g/dL
Platelets	195	144	150 – 500 /10^3/µL
Urea	48	56	17 – 43 /mg/dL
Creatinine	1.91	2.28	0.67 – 1.18 /mg/dL
AST	517	519	15 – 50 /U/L
ALT	499	472	3 – 45 /U/L
ALP	452	448	30 – 120 /U/L
GGT	443	485	< 55 /U/L
Total bilirubin	1.35	1.94	0.3 – 1.2 /mg/dL
Direct bilirubin	0.45	1.08	< 0.2 /mg/dL
LDH	782	768	100 – 247 /U/L
CK	322	318	10 – 171 /U/L
C-reactive protein	258.2	306.1	< 5.0 /mg/L
Procalcitonin	-	> 100	<0.50 /ng/mL
Glycated hemoglobin	-	6.5	4.0 – 6.0 /%

**Figure 2:**
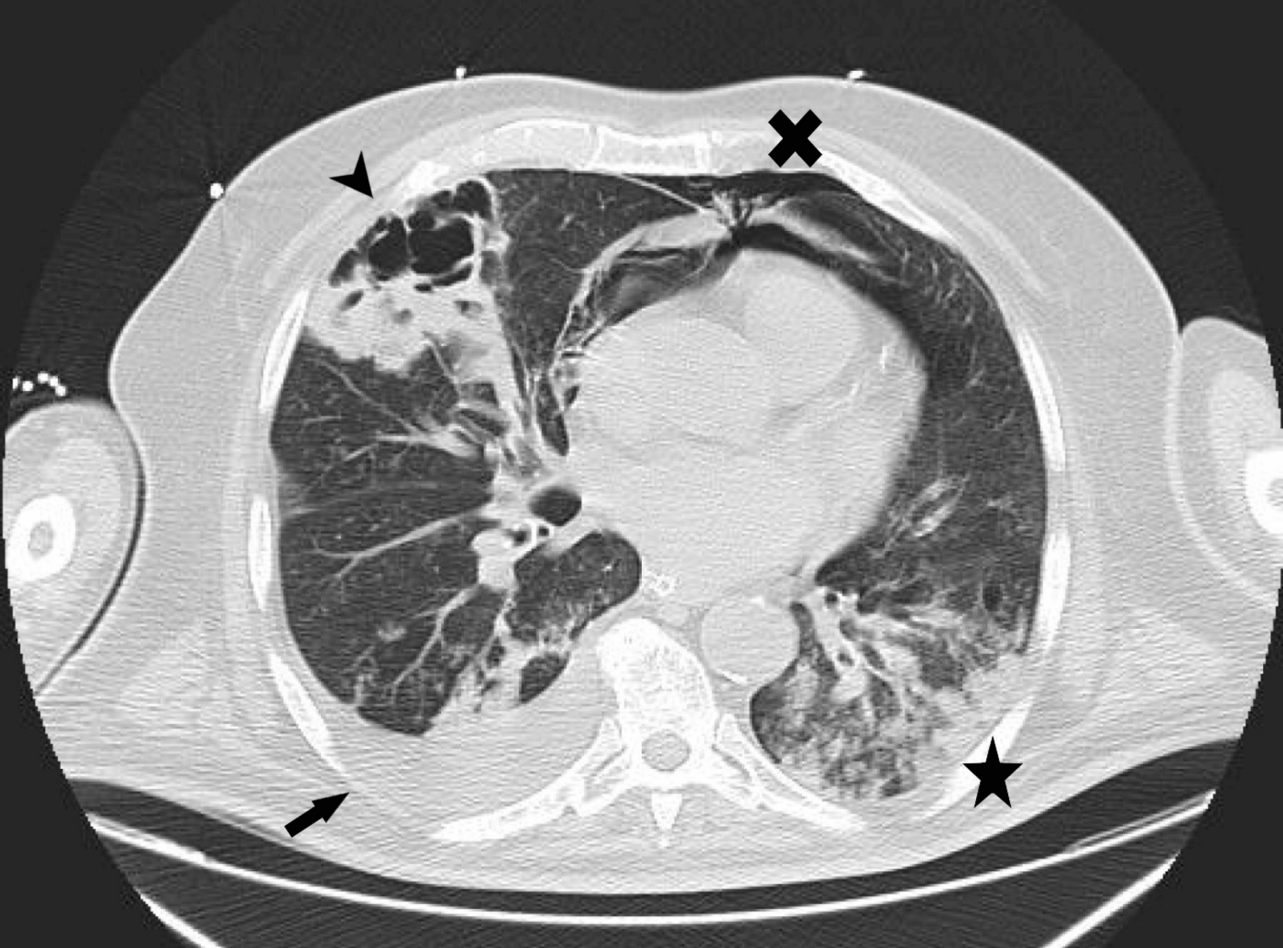
Axial CT scan of the patient's thorax. In the image, a cavitary lesion with thick, heterogeneous walls, suggestive of necrotizing pneumonia, is indicated by the arrowhead. The arrow points to a right-sided pleural effusion with passive atelectasis of the adjacent lung. Just below the cross, a pneumomediastinum is identified and the star indicates parenchymal infiltrates with areas of consolidation.

**Figure 3:**
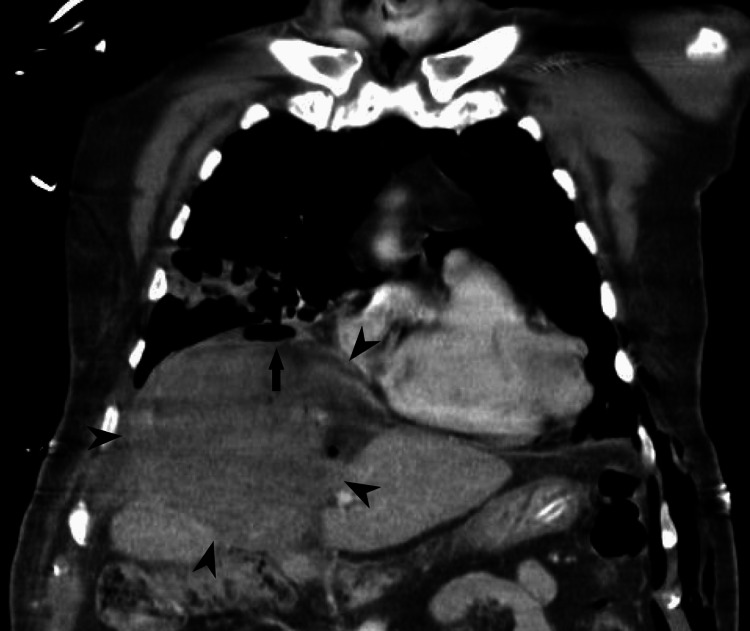
Coronal CT scan of the patient's thorax and upper abdomen. The image shows a large nodular formation in the upper region of the liver, measuring 110 mm in width, 134 mm in length, and 105 mm in height, with contrast-enhancing walls and an air-fluid level, suggestive of a hepatic abscess, marked by arrowheads. Continuity between the abdominal lesion and the pulmonary necrotizing process is noted, with diaphragmatic discontinuity, indicating diaphragmatic perforation secondary to the infectious process, marked by an arrow.

Antibiotic therapy was escalated to piperacillin/tazobactam and vancomycin, along with other supportive measures. A chest CT scan revealed a cavitated pulmonary lesion measuring 77 mm in length, 62 mm in width, and 60 mm in height, located in the anterior portion of the right upper lobe, extending into the middle lobe. The lesion had a thickened, heterogeneous wall, suggestive of an infectious etiology, likely necrotizing pneumonia. Additionally, bilateral parenchymal infiltrates were noted, predominantly in the lower lobes, with areas of consolidation. A right pleural effusion with passive atelectasis of the adjacent lung and pneumomediastinum was also present (Figure 2). The abdominal CT scan revealed a large nodular formation in the upper region of the liver, measuring 110 mm in width, 134 mm in length, and 105 mm in height, with contrast-enhancing walls and an air-fluid level, suggestive of a hepatic abscess. Continuity between the abdominal lesion and the pulmonary necrotizing process was noted, with diaphragmatic discontinuity, suggesting diaphragmatic perforation secondary to the infectious process (Figure 3).

**Figure 4:**
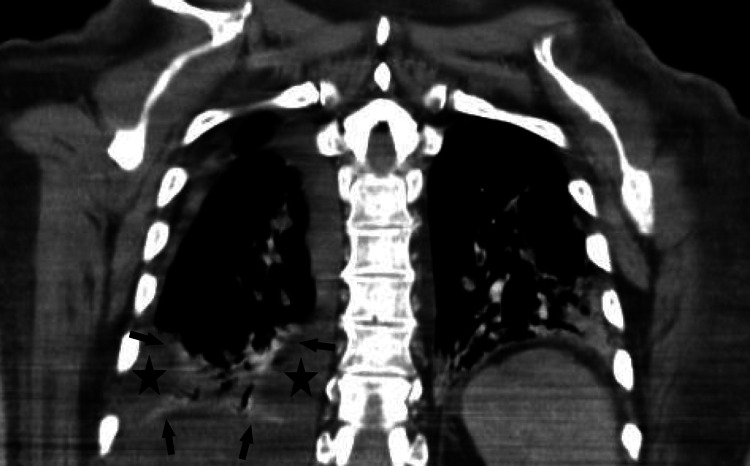
Coronal CT scan of the patient's thorax. The image shows a pleural effusion marked by the stars later confirmed to be a pleural empyema. The arrows indicate the boundary of the right lung.

Percutaneous drainage of the abscess was performed by Interventional Radiology. The purulent material was aspirated from the abscess for analysis, and a pigtail drain was inserted. Ultrasound-guided thoracentesis of the right pleural effusion yielded thick purulent fluid consistent with empyema (Figure 4) (fresh pH below the machine's measurable limit (pH < 6.8), LDH of 5921 U/L and glucose of 1.3 mg/dL) and a chest tube was placed.

**Figure 5:**
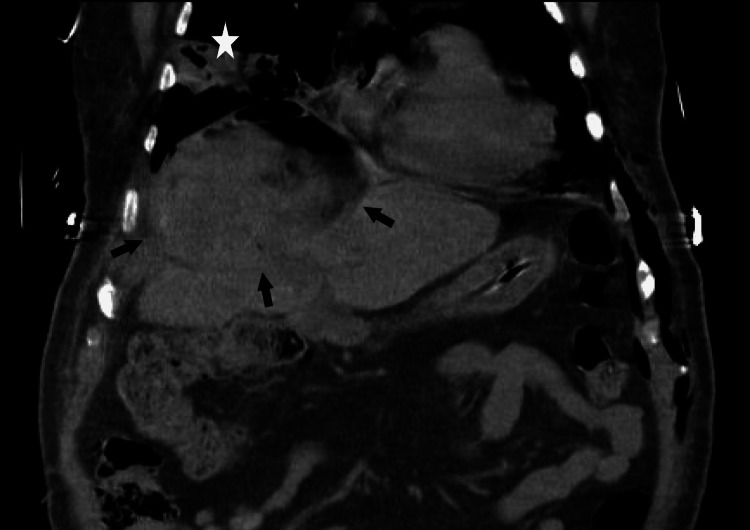
Coronal CT scan of the patient's lower thorax and upper abdomen. The image shows the liver abscess after percutaneous drainage with the placement of a pigtail catheter, marked by the arrows, displaying a slight reduction in volume and increased gas content within. The star indicates the necrotizing pneumonia, which is contiguous with the liver abscess, with visible diaphragmatic discontinuity.

**Figure 6:**
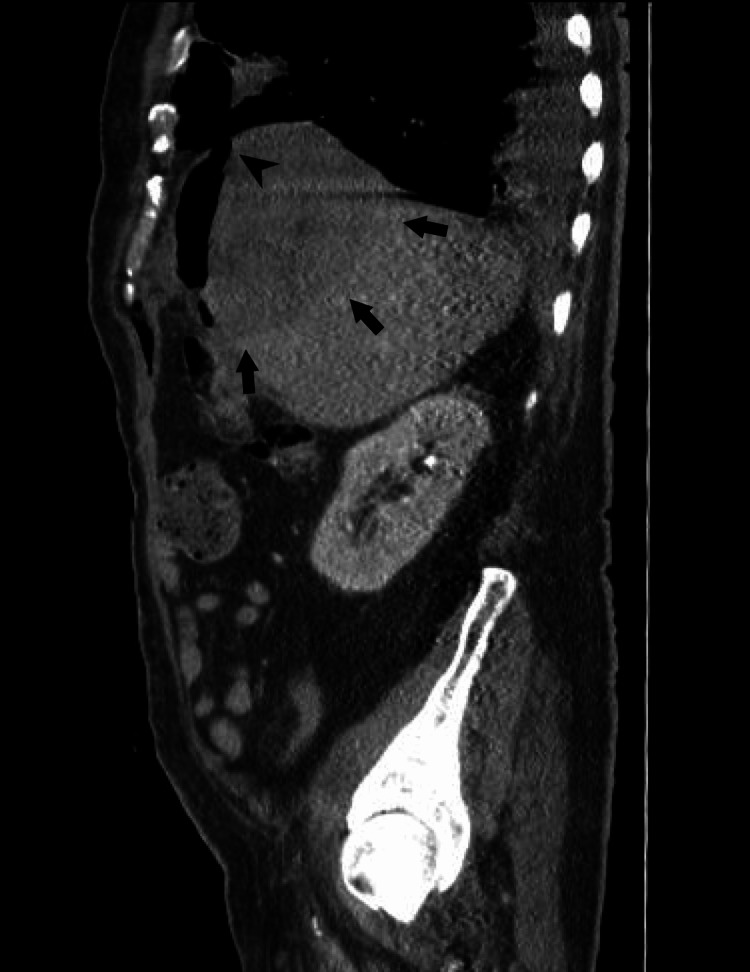
Sagittal CT scan of the patient's abdomen. The image shows a sagittal view of the liver abscess after percutaneous drainage, marked by arrows. The arrowhead indicates the diaphragmatic perforation.

**Figure 7:**
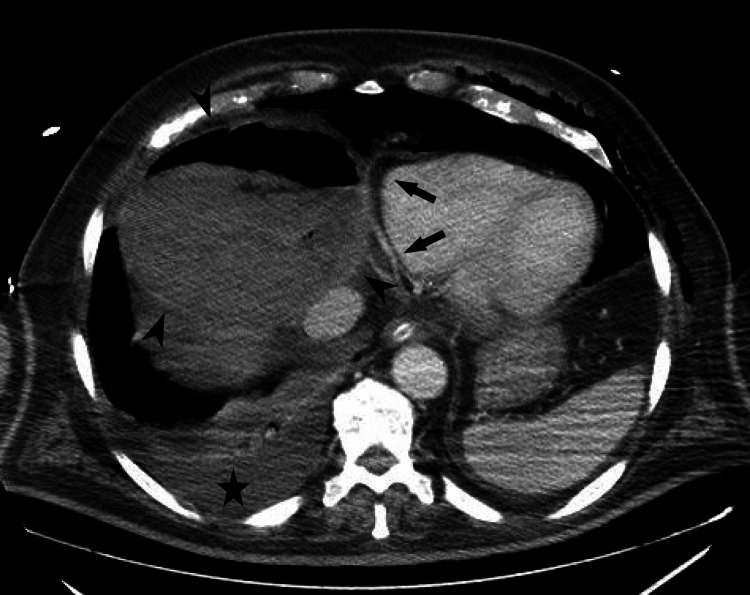
Axial CT scan showing the liver abscess (indicated by arrowheads), right pleural effusion (indicated by the star) and pericardial thickening (indicated by arrows).

Patient’s clinical condition continued to worsen, purulent material, similar to that aspirated from the hepatic abscess mixed with bile, was draining through the airway and the pigtail catheter at the hepatic abscess had low drainage volumes due to the thickness of the material. A CT scan was repeated which showed only a slight reduction in the abscess size (Figures 5, 6) and new mild pericardial thickening (Figure 7). After a multidisciplinary discussion, which included the Thoracic Surgery team from our referral hospital, surgical intervention for abscess drainage was performed by the General Surgery’s highly experienced team specialized in liver and biliary tract pathology.

Intraoperatively, a large hepatic abscess was identified and opened, revealing air and thick purulent material. A 35 mm irregular diaphragmatic perforation was observed, allowing communication with the right lung. Anticipating the technical impossibility of suturing an inflamed and friable diaphragm, they opted for a conservative strategy. Extensive irrigation of both the abscess cavity and abdominal cavity was performed, and the abscess cavity was packed with omentum to isolate it from the thoracic cavity. A large-caliber drain was left in the abscess cavity.

**Figure 8:**
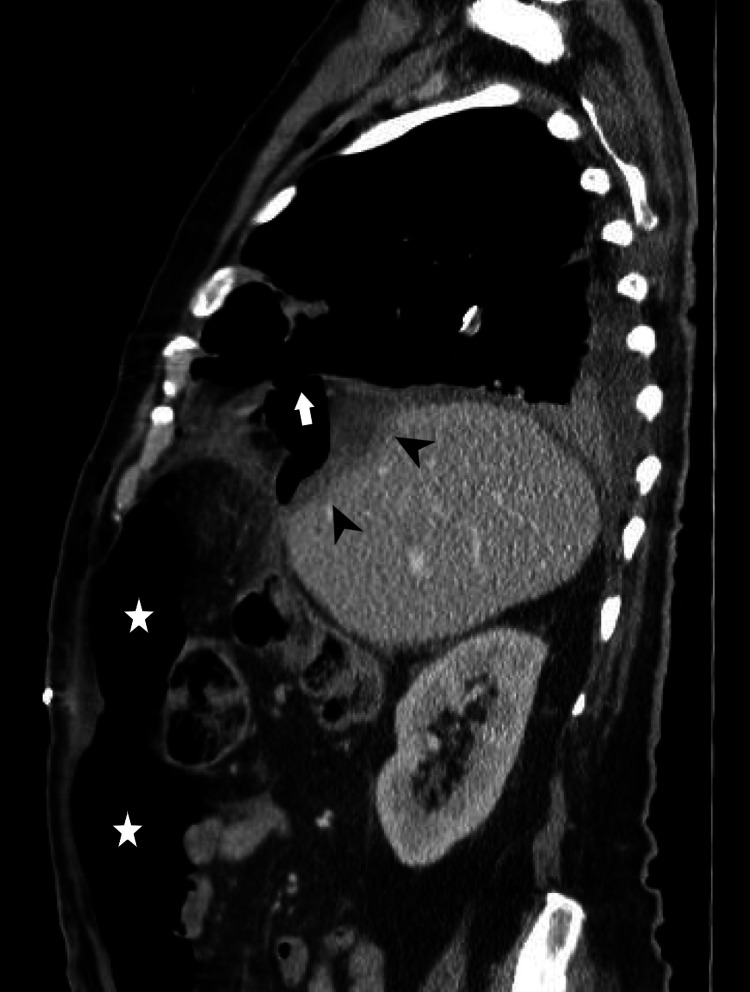
Sagittal CT scan of the patient's thorax and upper abdomen. The image displays a residual hepatic abscess following surgical drainage, indicated by the arrowheads. The arrow marks the diaphragmatic perforation, while the stars identify an extensive pneumoperitoneum.

A bronchoscopy was performed, which revealed a large amount of purulent material throughout the bronchial tree, which was sent for microbiological analysis. Despite the use of protective mechanical ventilation, continuous air drainage was observed during inspiration through the abdominal drain, and a follow-up CT scan revealed a large pneumoperitoneum (Figure 8). Nevertheless, the patient had constant monitoring of intra-abdominal pressure, which remained consistently low. Klebsiella pneumoniae was identified only in the hepatic abscess material, leading to the discontinuation of vancomycin. Tests for Entamoeba histolytica, HIV, and hepatitis were negative. Transthoracic echocardiography showed no abnormalities and transesophageal echocardiography was deemed unnecessary due to a very low suspicion of endocarditis.

The patient showed gradual improvement, leading to the discontinuation of invasive supportive measures and extubation on the twelfth postoperative day. At the time of extubation, the abdominal drain was still draining air; however, this ceased after extubation, with the pneumoperitoneum gradually subsiding. The patient completed a total of 21 days of piperacillin/tazobactam, followed by 14 days of amoxicillin/clavulanic acid, which was discontinued after favorable clinical and imaging progress. The abdominal drain was removed on the twenty-fourth day.

The patient was later transferred to the General Surgery department, where he underwent upper gastrointestinal endoscopy and colonoscopy which revealed no significant findings. He was discharged to a rehabilitation center and continued follow-up in the General Surgery outpatient clinic.

**Figure 9:**
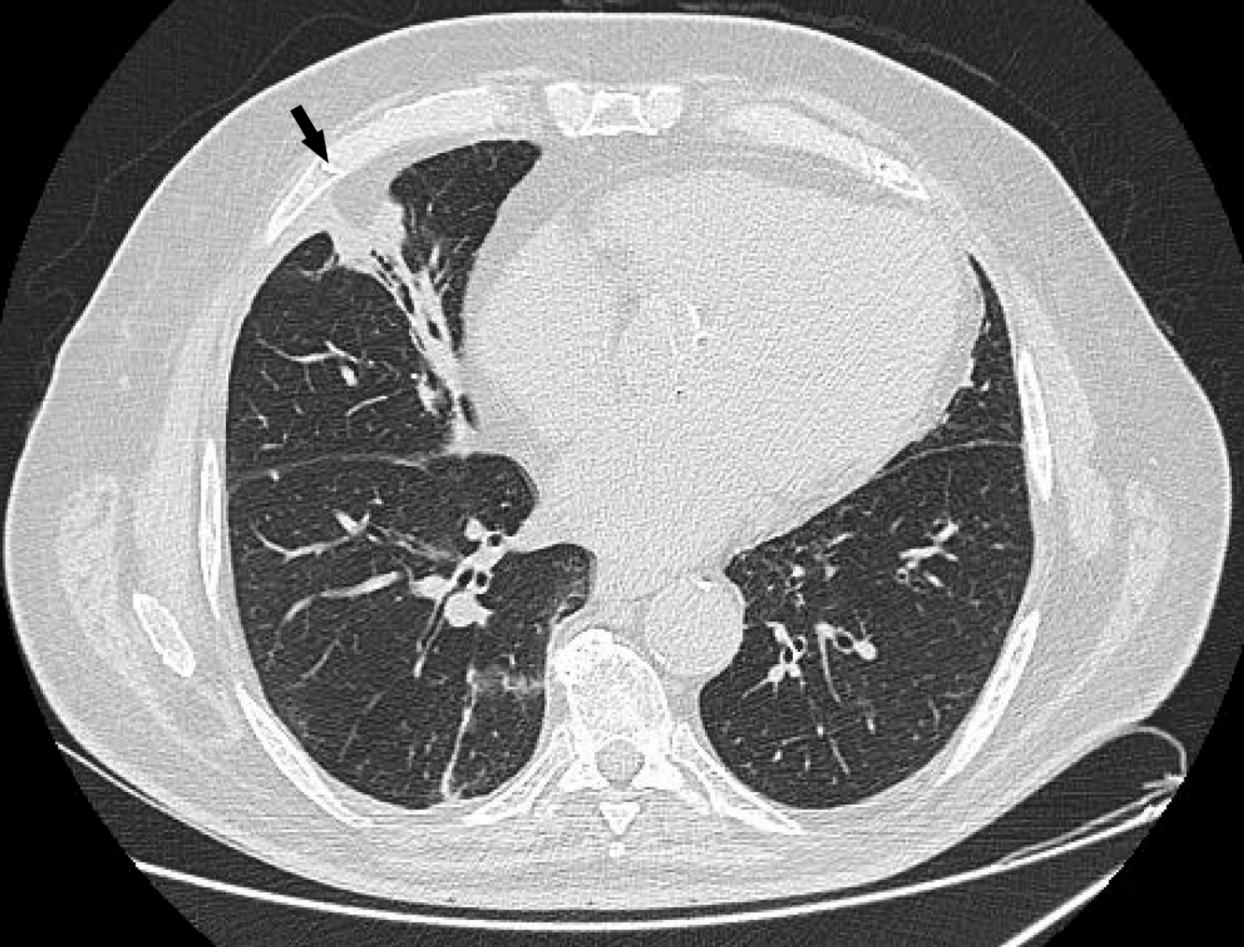
CT scan showing complete resolution of the necrotizing pneumonia, with only residual scarring remaining (indicated by the arrow).

**Figure 10:**
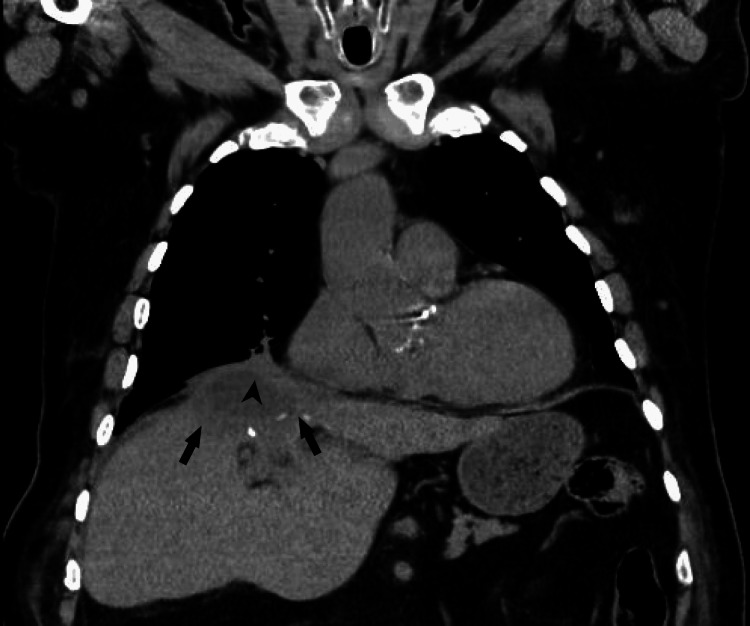
CT scan showing resolution of the hepatic abscess, with only residual scarring remaining (indicated by arrows), and complete cicatrization of the diaphragm (indicated by the arrowhead).

Notably, six months after discharge, imaging showed complete resolution of the necrotizing pneumonia and hepatic abscess, with only residual scarring remaining as evidence of the prior events (Figures 9, 10). Approximately two years after the hospitalization, the patient leads an active life without limitations and has undergone repeat endoscopic evaluations, which have shown no abnormalities.

## Discussion

A hepatic abscess is a rare condition that can present atypically, particularly in elderly patients [7]. The epidemiology, diagnosis, and treatment of PLAs have undergone significant changes in recent decades, with a rising incidence in older patients, now most frequently occurring in the fifth decade of life [7,8]. Despite advancements in management and a significant reduction in morbidity and mortality, PLAs remain a life-threatening condition [1,8,9].

The liver is particularly susceptible to the formation of PLAs for various reasons. The abscess may form through hematogenous spread, either via the hepatic artery, as in cases of septic shock with bacteremia, or through the portal vein, such as in diverticulitis or colorectal neoplasia [5,7]. Although hematogenous spread was traditionally considered rare, it is now recognized as a common etiology [3]. Additionally, biliary tract diseases, particularly ascending cholangitis in the context of lithiasis, neoplasia, or congenital disorders, as well as contiguous abdominal infections, can also be responsible for PLA formation [5,7]. Other known risk factors for PLA include type 2 diabetes mellitus, particularly with poor glycemic control, obesity [10], and hypertension [5]. Alcoholism is another risk factor, particularly related to Klebsiella pneumoniae [11].

Klebsiella pneumoniae is a Gram-negative bacterium that is part of the commensal flora of the nasal and gastrointestinal tracts, and it can occasionally cause community-acquired pneumonia and urinary tract infections [12]. Though rare, Klebsiella pneumoniae infection with PLAs can be associated with extrahepatic disease, including meningitis, endophthalmitis, otitis, spondylodiscitis, and even septic pulmonary embolism [11]. Unlike most PLAs, which are polymicrobial [13], these are more often monomicrobial [7]. The extra-pulmonary dissemination of the disease typically indicates greater severity and is referred to as invasive liver abscess syndrome (ILAS) [11,13]. While PLAs near the diaphragm can cause respiratory symptoms, they rarely cross the diaphragm, particularly in non-immunocompromised patients [14]. Diaphragmatic translocation or destruction with thoracic infection, resulting in hepatobronchial fistulas, pulmonary destruction, lung abscess, or pericarditis due to PLA contiguity, is extremely rare, with only a few cases reported in the literature [2,14,15].

Procalcitonin measurement can be useful, as its absolute value correlates with infection severity. It is also a marker of sepsis and generally appears to be higher in patients with PLAs and diabetes, suggesting that diabetic patients with PLAs are more susceptible to developing sepsis and septic shock [5]. Abdominal ultrasound is often used as a first-line diagnostic tool due to its availability, safety, and low cost [8]. However, recent studies recommend early CT scanning in suspected PLAs, because of its higher sensitivity and widespread availability, with MRI reserved for more specific indications [8,13]. The characterization of the abscess, particularly its size, is crucial. Regardless of age, gender, etiology, or risk factors, abscess size is an independent prognostic factor. Larger abscesses, particularly those exceeding 10 cm in diameter, are associated with a worse prognosis, longer hospital stays, higher in-hospital mortality, and a greater likelihood of extrahepatic disease [16].

The currently recommended first-line treatment is percutaneous drainage of the PLA, guided by imaging techniques, with surgery reserved for more particular cases. When combined with appropriate antibiotic therapy, this strategy appears to be safe and effective in most cases and should be initiated early [5,8,17,18]. In cases of percutaneous drainage failure, surgical intervention should be considered, as it is an effective strategy for controlling the infectious focus [17].

Diaphragmatic rupture is generally rare, most commonly occurring in high-energy trauma cases [19,20]. There are also clinical reports of spontaneous diaphragmatic rupture, particularly in the context of endometriosis [19]. The recommended treatment in such cases is typically surgery involving diaphragm suturing, which can be performed through either a thoracic or abdominal approach, determined on a case-by-case basis [19,20].

## Conclusions

This article presents the case of an elderly patient with a prolonged course of nonspecific symptoms, including abdominal pain, which subsequently developed respiratory complaints. This presentation suggests a primary PLA with subsequent pulmonary dissemination by contiguity. The nonspecific clinical presentation leads to a diagnosis delay, contributing to increased morbidity and higher risk of mortality. In this case, the first-line approach failed, necessitating surgical intervention, which confirmed the presence of a giant abscess and evidence of diaphragmatic perforation, in addition to necrotizing pneumonia, hepatobronchial fistula, and empyema. The authors found very limited scientific evidence in the literature regarding the best therapeutic approach for this specific situation. In this case, a conservative surgical strategy was used with success. Despite extensive local infection and systemic dissemination consistent with ILAS, as well as progression to septic shock requiring ICU admission, the patient achieved a gradual yet full recovery and ultimately returned to an active daily life. This was only possible due to the effective control of the infectious focus and supportive measures.
